# SARS-CoV-2 is less likely to infect aquatic food animals: sequence and phylogeny analysis of ACE2 in mammals and fish

**DOI:** 10.1186/s43556-020-00016-x

**Published:** 2020-11-20

**Authors:** Dong Chen, Yuchen Liu, Huihui Yang, Lisa Liu, Weiren Huang, Yongsheng Zhao

**Affiliations:** 1 Institute of Shenzhen Translational Medicine, Shenzhen Second People’s Hospital, the First Affiliated Hospital of Shenzhen University, Shenzhen, 518035 China; 2 Shenzhen Institutes of Advanced Technology, Chinese Academy of Sciences, Shenzhen, 518055 China; 3 School of Chemistry, Sun Yat-Sen University, Guangzhou, 510275 China

Dear Editor,

The ongoing coronavirus disease 2019 (COVID-19) global pandemic has a zoonotic origin, and was transmitted to humans via an undetermined intermediate host. There have been concerns that using aquatic animals as food may facilitate the transmission of the COVID-19 to humans. Recently, Beijing’s largest vegetable wholesale market was shut down after new domestic COVID-19 infections appeared and the novel coronavirus was detected on a chopping board used by a seller of imported salmon. This sparked speculations on whether the fish can spread the virus. A decreased in fish consumption has been reported in several countries and regions, in part for fear of the risk of viral transmission to humans.

COVID-19, which is caused by severe acute respiratory syndrome coronavirus 2 (SARS-CoV-2), has evolved into a global pandemic. Although SARS-CoV-2 was supposed to be a zoonotic source, with the closest sequence derived from the horseshoe bat [1], its transmission route to humans is unknown. SARS-CoV-2 infections have been reported in many mammals including dogs, cats, tigers, ferrets, macaques, and marmosets. Until now, no viruses that infect fish have been reported to pose a risk to human health [2]. The receptor binding domain (RBD) of the SARS-CoV-2 spike protein binds to the extracellular peptidase domain of angiotensin I converting enzyme 2 (ACE2) that mediates cell entry. ACE2 is widely expressed in vertebrates, and its sequence is highly conserved across mammals, suggesting that SARS-CoV-2 could use orthologues of ACE2 for cell entry.

In this study, we describe a sequence comparison in ACE2s from a broad range of species. The percentages of amino acid sequence identities were calculated from the alignment of ACE2s of the selected vertebrates (Fig. 1a). Among all ACE2s, human share a sequence identity of ~ 80% or greater with other mammals. Human ACE2 shows a high degree of similarity to the reported amino acid sequences of ACE2 of the potential reservoir host–bat (81.4%) and the potential intermediate host–pangolin (84.7%). However, the amino acid sequence of human ACE2 shows a relatively low sequence identity with that of birds, amphibians, reptiles, and fish. The sequence identities between human ACE2 and the 41 fish ACE2s range from 55.1% (swamp eel) to 60.5% (coelacanth).

**Fig. 1:**
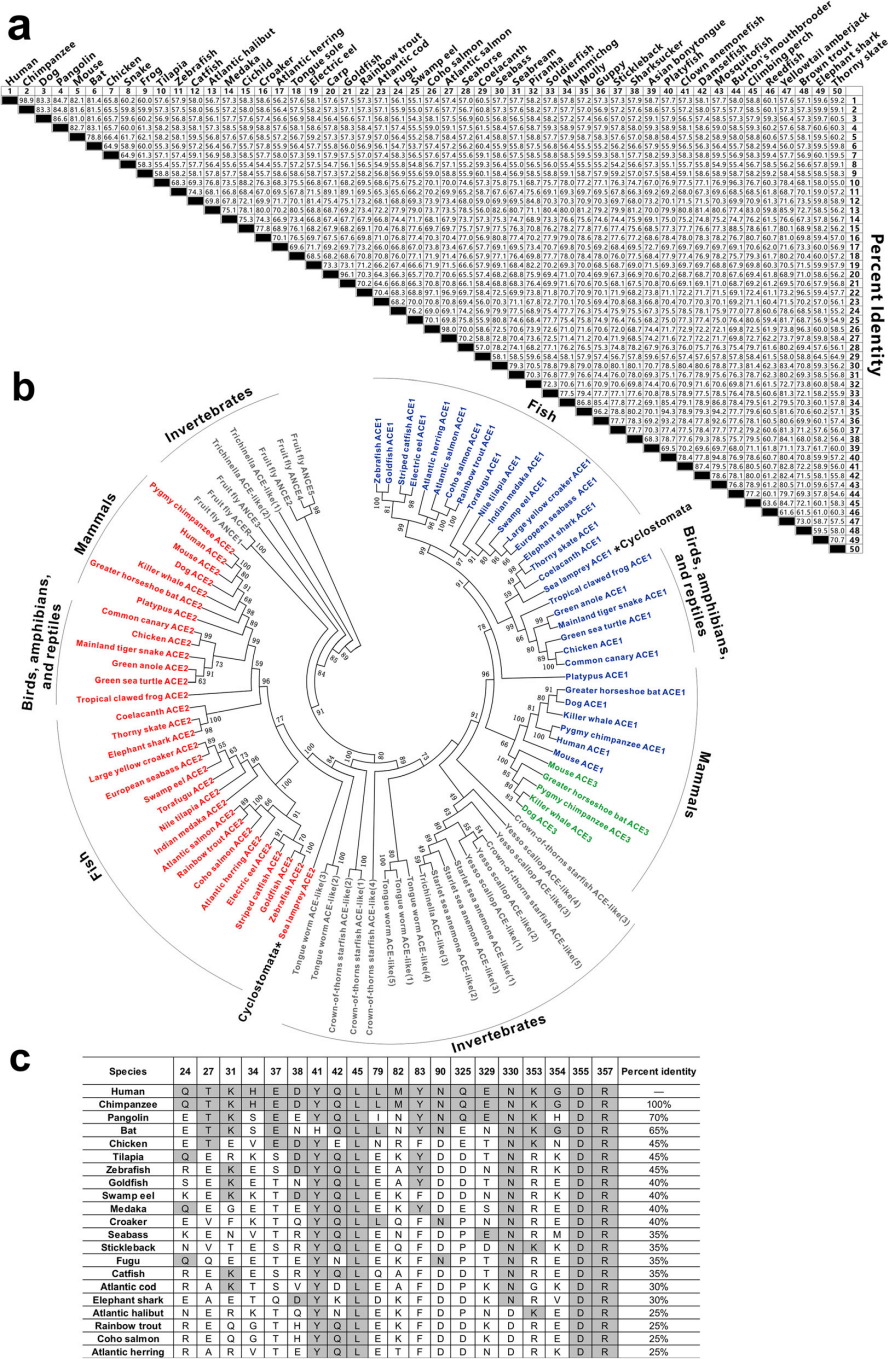
Sequence comparison and phylogenetic analysis of ACE2 and its homologues among diverse species. **a** The identities of ACE2 amino acid sequences from a list of vertebrates. **b** Phylogenetic analysis of ACE2 and its homologues from representative species. The phylogenetic tree was constructed by the maximum-likelihood method using the MEGA 7.0 software, based on the alignments of the amino acid sequences of ACEs using Clustalx 1.83 program. The number shown at each branch node indicates the bootstrap value (%) estimated by 1000 replications. **c** Comparison of the interaction sites of ACE2 in contact with SARS-CoV-2 among vertebrates. Amino acid sequence alignment of ACE2s was generated using Clustalx1.83 software. Conserved amino acids are shaded in gray. The sequences and their respective sources of ACEs are listed in Supplementary Table 1

ACE2 and its homologues are widely expressed in both vertebrates and invertebrates. There are three types of ACE in most of mammals, two types of ACE in non-mammalian vertebrates, and three or more types of ACE homolog in invertebrates (Fig. 1b). Invertebrates have derived more types of ACE-like proteins, suggesting that this may be the ancestral condition in evolution. Interestingly, ACE3 was found in mammals (pseudogene in human) but not in other vertebrates through database searching, indicating that ACE3 may has been lost in several major lineages independently. The phylogenetic tree shows the corresponding evolutionary relationship of the ACE1 and ACE2 sequences from divergent animals. According to the phylogeny tree analysis, mammalian ACE2s and fish ACE2s were clustered into different branches. The amino acid sequences of ACE2 in the Atlantic salmon and coho salmon, which were suspected of being contaminative by SARS-CoV-2, are phylogenetically further away from human (Fig. 1b). The low genetic similarity of ACE2s between human and fish, therefore, negates the possibility of the virus infecting aquatic food animals.

It has been reported that a total of 20 amino acid residues of human ACE2 are responsible for the binding with SARS-CoV [3]. Analysis of the receptor binding motif, a portion of the RBD that makes contact with ACE2, revealed that most amino acid residues essential for ACE2 binding by SARS-CoV are conserved in SARS-CoV-2 [4]. The 20 interaction sites of ACE2 in contact with SARS-CoV-2 were compared between human and fish (Fig. 1c). Out of 20 amino acid residues involved in the direct interaction, 3 of them are shared by all 21 species analyzed. It is interesting to note that human ACE2 shares 70% and 65% of conserved contacting amino acid residues with pangolin and bat ACE2, respectively (Fig. 1c). Comparing the binding sites between human and fish ACE2s showed that only 25 ~ 45% of the contacting amino acid residues are conserved, suggesting that this may be an obstacle in the binding between SARS-CoV-2 S-protein and fish ACE2s. Recently, Damas et al. performed a structural analysis of the ACE2/SARS-CoV-2 S-binding interface by utilizing a dataset of ACE2 sequences from 410 vertebrate species, and revealed that only mammals were at high risk for SARS-CoV-2 infection [5]. Future research on molecular modelling of different ACE2s and its homologues from both vertebrates and invertebrates, and their docking with SARS-CoV-2 S-protein structure and subsequent molecular interaction analysis will be more persuasive and valuable.

In summary, we describe a comparison in ACE2 and its homologues from a broad range of vertebrates and invertebrates. Several lines of evidence containing sequence identity, phylogenetic analysis, and comparison of interaction sites of ACE2s among vertebrates, show that SARS-CoV-2 may infect a broad range of mammals, but few fish. Thus, based on the current knowledge and supporting materials, there is no evidence to suggest that the novel coronavirus SARS-CoV-2 can infect aquatic food animals.

## Supplementary Information

**Additional file 1: Supplementary Information for Materials and Methods**. **Supplementary Table 1**. List of protein sequences of ACE2 and its homologues used in this study.

## Data Availability

The protein sequences of ACE2 and its homologues used in this study are provided in the Supplementary Table 1.

## Abbreviations

ACE2: Angiotensin I converting enzyme 2
COVID-19: Coronavirus disease 2019
RBD: Receptor binding domain
SARS-CoV-2: Severe acute respiratory syndrome coronavirus 2
