# Supplementary material for: SARS-CoV-2 is less likely to infect aquatic food animals: sequence and phylogeny analysis of ACE2 in mammals and fish
Source: Mol Biomed. 2020 Nov 20;1:13. doi: 10.1186/s43556-020-00016-x (PMC7676880; doi:10.1186/s43556-020-00016-x)
Supplement: Supplementary file 1 — Additional file 1: Supplementary Information for Materials and Methods. Supplementary Table 1. List of protein sequences of ACE2 and its homologues used in this study. [file 43556_2020_16_MOESM1_ESM.pdf]

Molecular Biomedicine

Supplementary Data

SARS-CoV-2 is less likely to infect aquatic food animals: Sequence and phylogeny analysis of ACE2 in mammals and fish

Dong Chen[1,2], Yuchen Liu[1], Huihui Yang[3], Lisa Liu[1], Weiren Huang[1,*], Yongsheng Zhao[1,*]

[1] Institute of Shenzhen Translational Medicine, Shenzhen Second People's Hospital, the First Affiliated Hospital of Shenzhen University, Shenzhen 518035, China

[2] Shenzhen Institutes of Advanced Technology, Chinese Academy of Sciences, Shenzhen 518055, China

[3] School of Chemistry, Sun Yat-Sen University, Guangzhou 510275, China

* Corresponding Author: Weiren Huang and Yongsheng Zhao

Email address: pony8980@163.com (W. Huang), zhaoyongsheng@188.com (Y. Zhao).

Supplementary Information for Materials and Methods

The publicly available amino acid sequences of ACE and its homologues were retrieved from the NCBI database, UniProt database, and Ensemble genome browser. Alignments of amino acid sequences of ACEs were generated using Clustalx1.83 software (Plate-Forme de Bio-Informatique, Illkirch Cedex, France). The sequence identities were calculated using MegAlign of the DNAstar software package (DNASTAR, Inc., Madison, WI, USA). Phylogenetic trees were constructed by the maximum-likelihood method using the MEGA 7.0 software and bootstrap values (%) were calculated from 1,000 replications to estimate the robustness of internal branches.

Supplementary Table 1. List of protein sequences of ACE2 and its homologues used in this study.

Receptor	Common Name	Latin name	Source	Accession
ACE1	Human	*Homo sapiens*	NCBI	NP_000780.1
ACE1	Pygmy chimpanzee	*Pan paniscus*	NCBI	XP_034798573.1
ACE1	Mouse	*Mus musculus*	NCBI	NP_997507.1
ACE1	Dog	*Canis lupus familiaris*	NCBI	XP_003639297.1
ACE1	Greater horseshoe bat	*Rhinolophus ferrumequinum*	NCBI	XP_032945085.1
ACE1	Killer whale	*Orcinus orca*	NCBI	XP_004275705.1
ACE1	Platypus	*Ornithorhynchus anatinus*	NCBI	XP_028931563.1
ACE1	Chicken	*Gallus gallus*	NCBI	NP_001161204.1
ACE1	Common canary	*Serinus canaria*	NCBI	XP_009094827.1
ACE1	Green anole	*Anolis carolinensis*	NCBI	XP_008111340.1
ACE1	Mainland tiger snake	*Notechis scutatus*	NCBI	XP_026535627.1
ACE1	Green sea turtle	*Chelonia mydas*	NCBI	XP_027680218.1
ACE1	Tropical clawed frog	*Xenopus tropicalis*	NCBI	NP_001116882.1
ACE1	Zebrafish	*Danio rerio*	NCBI	XP_694336.5
ACE1	Striped catfish	*Pangasianodon hypophthalmus*	NCBI	XP_026771009.1
ACE1	Swamp eel	*Monopterus albus*	NCBI	XP_020462022.1
ACE1	Coho salmon	*Oncorhynchus kisutch*	NCBI	XP_031655455.1
ACE1	Rainbow trout	*Oncorhynchus mykiss*	Ensemble	ENSOMYT00000021787.1
ACE1	Electric eel	*Electrophorus electricus*	NCBI	XP_026877702.2
ACE1	Atlantic salmon	*Salmo salar*	Ensemble	ENSSSAT00000097927.1
ACE1	Torafugu	*Takifugu rubripes*	NCBI	XP_011602426.2
ACE1	Atlantic herring	*Clupea harengus*	NCBI	XP_012672791.2
ACE1	Large yellow croaker	*Larimichthys crocea*	NCBI	XP_010734599.3
ACE1	Indian medaka	*Oryzias melastigma*	NCBI	XP_024141540.1
ACE1	Coelacanth	*Latimeria chalumnae*	NCBI	XP_014345475.1
ACE1	Nile tilapia	*Oreochromis niloticus*	NCBI	XP_003438793.1
ACE1	Goldfish	*Carassius auratus*	NCBI	XP_026133022.1
ACE1	European seabass	*Dicentrarchus labrax*	Ensemble	ENSDLAT00005062542.1
ACE1	Elephant shark	*Callorhinchus milii*	NCBI	XP_007905531.1
ACE1	Thorny skate	*Amblyraja radiata*	NCBI	XP_032891034.1
ACE1	Sea lamprey	*Petromyzon marinus*	NCBI	XP_032807619.1
ACE2	Human	*Homo sapiens*	NCBI	NP_001358344.1
ACE2	Pygmy chimpanzee	*Pan paniscus*	NCBI	XP_008972428.2
ACE2	Rhesus monkey	*Macaca mulatta*	NCBI	NP_001129168.1
ACE2	Mouse	*Mus musculus*	NCBI	NP_001123985.1
ACE2	Dog	*Canis lupus familiaris*	NCBI	NP_001158732.1
ACE2	Greater horseshoe bat	*Rhinolophus ferrumequinum*	Ensemble	ENSRFET00010020887.1
ACE2	Malayan pangolin	*Manis javanica*	NCBI	XP_017505752.1
ACE2	Killer whale	*Orcinus orca*	NCBI	XP_033283819.1
ACE2	Platypus	*Ornithorhynchus anatinus*	Ensemble	ENSOANT00000004084.3
ACE2	Chicken	*Gallus gallus*	NCBI	XP_416822
ACE2	Common canary	*Serinus canaria*	NCBI	XP_009087922.1
ACE2	Green anole	*Anolis carolinensis*	NCBI	XP_008105456.1
ACE2	Mainland tiger snake	*Notechis scutatus*	NCBI	XP_026530754.1
ACE2	Green sea turtle	*Chelonia mydas*	NCBI	XP_007070561.1
ACE2	Tropical clawed frog	*Xenopus tropicalis*	NCBI	XP_002938293.2
ACE2	Zebrafish	*Danio rerio*	NCBI	XP_005169417.1
ACE2	Striped catfish	*Pangasianodon hypophthalmus*	NCBI	XP_026803610.1
ACE2	Swamp eel	*Monopterus albus*	NCBI	XP_020465646.1
ACE2	Coho salmon	*Oncorhynchus kisutch*	Ensemble	ENSOKIT00005063409.1
ACE2	Rainbow trout	*Oncorhynchus mykiss*	NCBI	XP_021433278.1
ACE2	Electric eel	*Electrophorus electricus*	NCBI	XP_026867211.1
ACE2	Atlantic salmon	*Salmo salar*	Ensemble	ENSSSAT00000116573.1
ACE2	Torafugu	*Takifugu rubripes*	NCBI	XP_029702274.1
ACE2	Atlantic herring	*Clupea harengus*	Ensemble	ENSCHAT00000054726.1
ACE2	Large yellow croaker	*Larimichthys crocea*	NCBI	XP_010730146.1
ACE2	Indian medaka	*Oryzias melastigma*	NCBI	XP_024150631.1
ACE2	Coelacanth	*Latimeria chalumnae*	NCBI	XP_005997915.2

ACE2	Nile tilapia	*Oreochromis niloticus*	NCBI	XP_003445853.2
ACE2	Goldfish	*Carassius auratus*	NCBI	XP_026131313.1
ACE2	Tiger tail seahorse	*Hippocampus comes*	NCBI	XP_019742561.1
ACE2	Platyfish	*Xiphophorus maculatus*	Ensemble	ENSXMAT00000001990.2
ACE2	Bicolor damselfish	*Stegastes partitus*	NCBI	XP_008290762.1
ACE2	Reedfish	*Erpetoichthys calabaricus*	NCBI	XP_028655640.1
ACE2	Clown anemonefish	*Amphiprion ocellaris*	NCBI	XP_023124156.1
ACE2	Climbing perch	*Anabas testudineus*	NCBI	XP_026233431.1
ACE2	Yellowtail amberjack	*Seriola lalandi*	Ensemble	ENSSLDT00000031369.1
ACE2	Live sharksucker	*Echeneis naucrates*	UniProt	A0A665VWQ8
ACE2	Asian bonytongue	*Scleropages formosus*	Ensemble	ENSSFOT00015015136.2
ACE2	Spotted gar	*Lepisosteus oculatus*	NCBI	XP_006639185.1
ACE2	Stickleback	*Gasterosteus aculeatus*	Ensemble	ENSGACT00000019964.1
ACE2	Brown trout	*Salmo trutta*	Ensemble	ENSSTUT00000072447.1
ACE2	Western mosquitofish	*Gambusia affinis*	Ensemble	ENSGAFT00000012716.1
ACE2	Burton's mouthbrooder	*Haplochromis burtoni*	NCBI	XP_005943362.1
ACE2	Guppy	*Poecilia reticulata*	NCBI	XP_008402714.1
ACE2	European seabass	*Dicentrarchus labrax*	Ensemble	ENSDLAT00005056141.1
ACE2	Amazon molly	*Poecilia formosa*	NCBI	XP_007560208.1
ACE2	Pinecone soldierfish	*Myripristis murdjan*	NCBI	XP_029904152.1
ACE2	Mummichog	*Fundulus heteroclitus*	NCBI	XP_021178198.1
ACE2	Gilthead seabream	*Sparus aurata*	Ensemble	ENSSAUT00010042842.1
ACE2	Atlantic halibut	*Hippoglossus hippoglossus*	NCBI	XP_034437236.1
ACE2	Flier cichlid	*Archocentrus centrarchus*	NCBI	XP_030582139.1
ACE2	Tongue sole	*Cynoglossus semilaevis*	NCBI	XP_016887914.1
ACE2	Common carp	*Cyprinus carpio*	NCBI	KTF89199.1
ACE2	Red-bellied piranha	*Pygocentrus nattereri*	NCBI	XP_017550079.1
ACE2	Atlantic cod	*Gadus morhua*	NCBI	XP_030232530.1
ACE2	Elephant shark	*Callorhinchus milii*	NCBI	XP_007889845.1
ACE2	Thorny skate	*Amblyraja radiata*	NCBI	XP_032888812.1
ACE2	Sea lamprey	*Petromyzon marinus*	NCBI	XP_032835032.1
ACE3	Pygmy chimpanzee	*Pan paniscus*	NCBI	XP_008962042.2
ACE3	Mouse	*Mus musculus*	NCBI	XP_006533116.1
ACE3	Dog	*Canis lupus familiaris*	NCBI	XP_022278528.1
ACE3	Greater horseshoe bat	*Rhinolophus ferrumequinum*	NCBI	XP_032944961.1
ACE3	Killer whale	*Orcinus orca*	NCBI	XP_012390484.2
ACE-like(1)	Starlet sea anemone	*Nematostella vectensis*	NCBI	XP_001626163.2
ACE-like(2)	Starlet sea anemone	*Nematostella vectensis*	NCBI	XP_032231453.1
ACE-like(3)	Starlet sea anemone	*Nematostella vectensis*	NCBI	XP_001630010.1
ACE-like(1)	Trichinella	*Trichinella papuae*	NCBI	KRZ79474.1
ACE-like(2)	Trichinella	*Trichinella papuae*	NCBI	KRZ80967.1
ACE-like(3)	Trichinella	*Trichinella papuae*	NCBI	KRZ80965.1
ACE-like(1)	Crown-of-thorns starfish	*Acanthaster planci*	NCBI	XP_022087764.1
ACE-like(2)	Crown-of-thorns starfish	*Acanthaster planci*	NCBI	XP_022085293.1
ACE-like(3)	Crown-of-thorns starfish	*Acanthaster planci*	NCBI	XP_022084568.1
ACE-like(4)	Crown-of-thorns starfish	*Acanthaster planci*	NCBI	XP_022079667.1
ACE-like(5)	Crown-of-thorns starfish	*Acanthaster planci*	NCBI	XP_022084566.1
ACE-like(1)	Yesso scallop	*Mizuhopecten yessoensis*	NCBI	XP_021364008.1
ACE-like(2)	Yesso scallop	*Mizuhopecten yessoensis*	NCBI	XP_021341680.1
ACE-like(3)	Yesso scallop	*Mizuhopecten yessoensis*	NCBI	XP_021364003.1
ACE-like(4)	Yesso scallop	*Mizuhopecten yessoensis*	NCBI	XP_021364030.1
ANCE1	Fruit fly	*Drosophila melanogaster*	Ensemble	FBtr0080553
ANCE2	Fruit fly	*Drosophila melanogaster*	Ensemble	FBtr0080554
ANCE3	Fruit fly	*Drosophila melanogaster*	Ensemble	FBtr0343641
ANCE4	Fruit fly	*Drosophila melanogaster*	Ensemble	FBtr0088664
ANCE5	Fruit fly	*Drosophila melanogaster*	Ensemble	FBtr0072420
ACER	Fruit fly	*Drosophila melanogaster*	Ensemble	FBtr0333416
ACE-like(1)	Tongue worm	*Saccoglossus kowalevskii*	NCBI	XP_006816593.1
ACE-like(2)	Tongue worm	*Saccoglossus kowalevskii*	NCBI	XP_002735388.2
ACE-like(3)	Tongue worm	*Saccoglossus kowalevskii*	NCBI	XP_006817325.1
ACE-like(4)	Tongue worm	*Saccoglossus kowalevskii*	NCBI	XP_006823284.1
ACE-like(5)	Tongue worm	*Saccoglossus kowalevskii*	NCBI	XP_002741143.2
